# Supplementary material for: Direct observation of exceptional points in coupled photonic-crystal lasers with asymmetric optical gains
Source: Nat Commun. 2016 Dec 21;7:13893. doi: 10.1038/ncomms13893 (PMC5187586; doi:10.1038/ncomms13893)
Supplement: Supplementary Information — Supplementary figures, supplementary note and supplementary references. [file ncomms13893-s1.pdf]

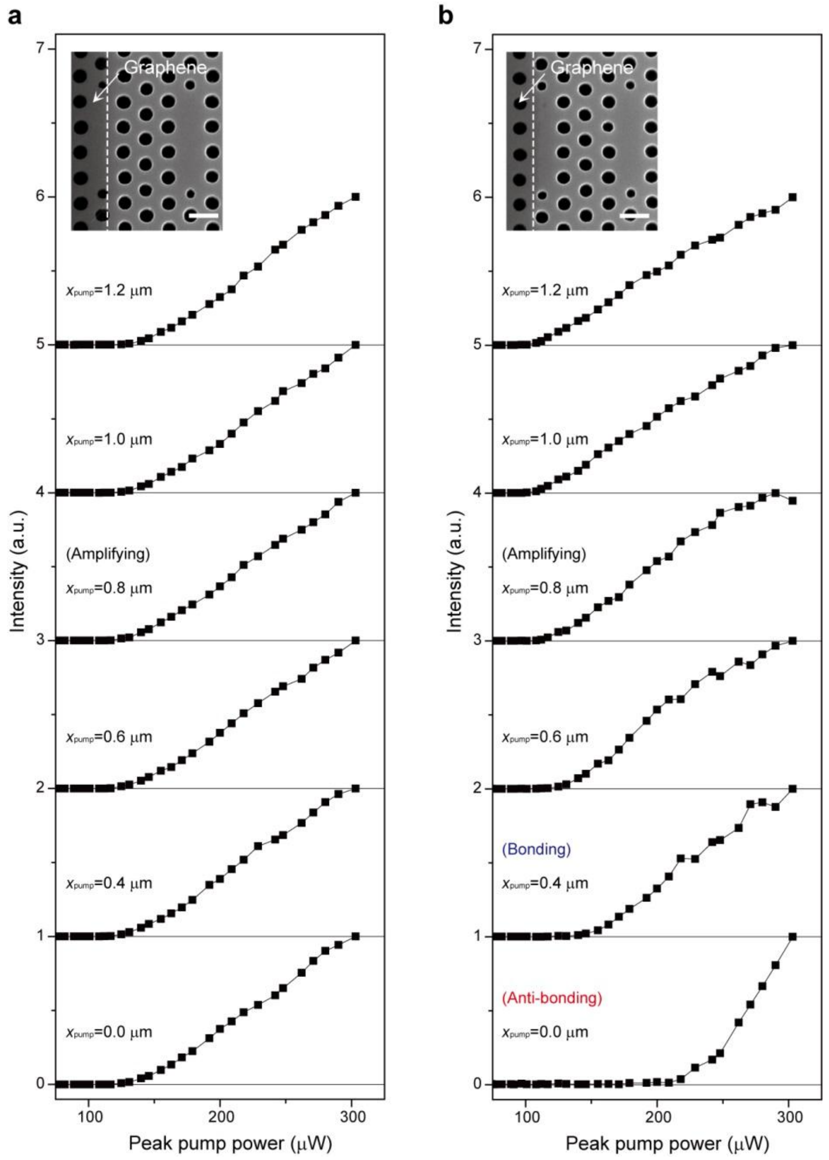

Supplementary Figure 1 | Measured L–L curves in the coupled PhC cavities with graphene. (**a**) Measured output intensities of the single lasing peak in the coupled cavities with large-area graphene (Fig. 3a) as a function of the peak pump power at x_{pump} = 1.2, 1.0, 0.8, 0.6, 0.4 and 0.0 μm (top to bottom of panel). All lasing thresholds were ~125 μW. Similar lasing thresholds at different pump positions exhibit a truly single-mode lasing operation (amplifying mode). Inset, fabricated laser structure, which is the same as that in the inset of Fig. 3a. Scale bar, 500 nm. (**b**) Measured output intensities of the lasing peak at ~1505 nm in the coupled cavities with small-

area graphene (black lines in Fig. 3f) as a function of peak pump power at x_{pump} = 1.2, 1.0, 0.8, 0.6 and 0.4 μm (top to bottom of panel). Lasing thresholds were ~110 μW at x_{pump} = 1.2, 1.0 and 0.8 μm. At x_{pump} = 0.6 and 0.4 μm, the lasing thresholds slightly increased up to ~126 μW and 140 μW, respectively. The bottom of the panel (x_{pump} = 0.0 μm) shows the measured output intensities of the lasing peak at ~1510 nm (red lines in Fig. 3f): the lasing threshold was ~220 μW. The anti-bonding, bonding and amplifying lasing modes were excited at x_{pump} = 0.0, 0.4 and ≥ 0.8 μm, respectively. Inset, fabricated laser structure, which is the same as that in the inset of Fig. 3f. Scale bar, 500 nm.

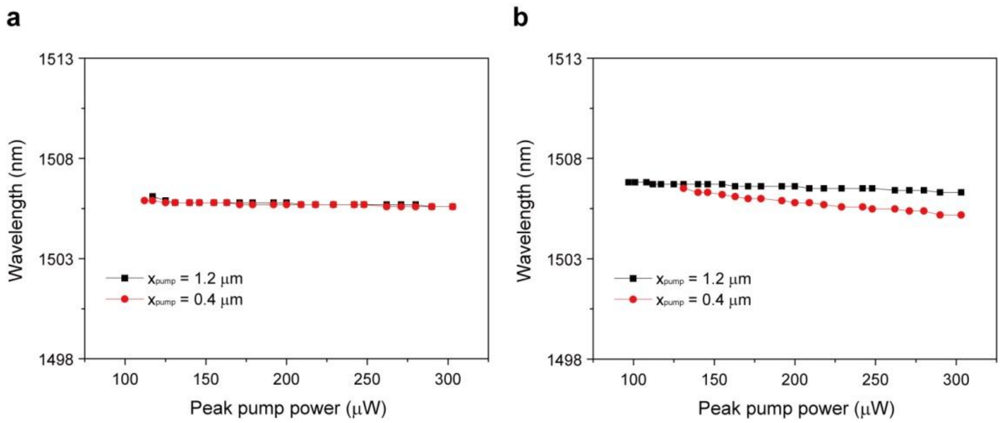

Supplementary Figure 2 | Lasing wavelength vs. peak pump power in the coupled PhC cavities with graphene. (**a**) Measured lasing wavelengths as a function of the peak pump power in the coupled cavities with large-area graphene (Fig. 3a) at x_{pump} = 1.2 (black line; amplifying mode) and 0.4 μm (red line; amplifying mode). The wavelength shifts for both pump positions were less than 0.5 nm. (**b**) Measured lasing wavelengths as a function of the peak pump power in the coupled cavities with small-area graphene (Fig. 3f) at x_{pump} = 1.2 (black line; amplifying mode) and 0.4 μm (red line; bonding mode). The wavelength shift for the amplifying mode was less than 0.5 nm, whereas the shift of the bonding mode was ~1.5 nm.

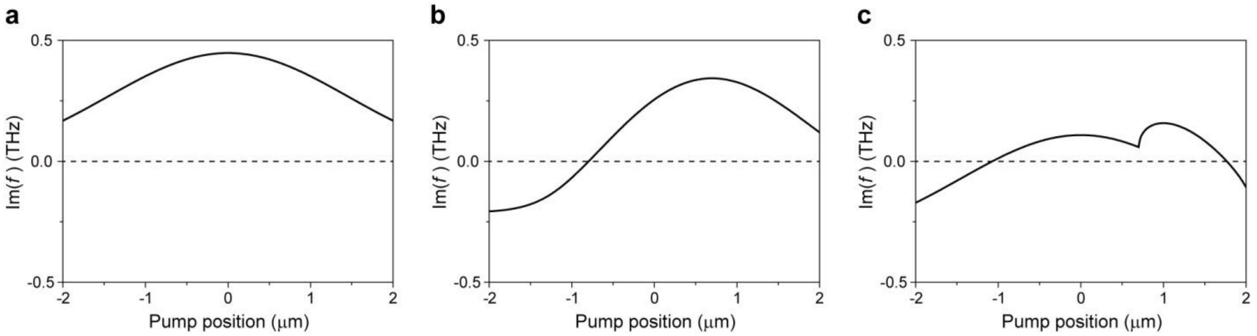

Supplementary Figure 3 | Calculated Im(*f*) of supermodes. (**a**–**c**) Calculated Im(*f*) using Eq. 1, in the coupled PhC cavities with no graphene (**a**), large-area graphene (**b**) and small-area graphene (**c**). The cavity structures of Fig. 4a–c were employed in these calculations. The pump positions for Im(*f*) > 0 are approximately $-2 < x_{\mathrm{pump}} < 2$ μm (**a**), $-1 < x_{\mathrm{pump}} < 2$ μm (**b**) and $-1 < x_{\mathrm{pump}} < 2$ μm (**c**), which agree well with the positions of the measured lasing peaks in Figs. 2 and 3.

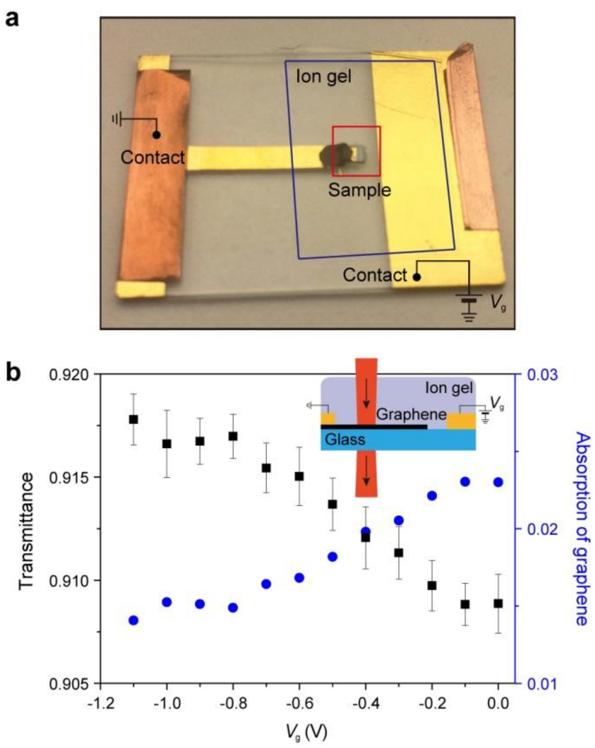

Supplementary Figure 4 | Tuning of optical loss of graphene. (**a**) Photograph of the fabricated device of Fig. 5a. The sample including the coupled PhC cavities with partially covered graphene (red rectangle) was placed between the two large Ti/Au contacts on the glass substrate, and was electrically connected to the left T-shaped contact using carbon paste. Ion gel (blue rectangle) fully covered the sample and the two contacts. The gap between the two contacts is small enough (~1 mm) to apply strong electric fields to the sample[1,2]. (**b**) Measured transmittance (left *y*-axis, black squares) of normally incident light through the glass/graphene/ion gel structure (inset), as a function of gate voltage V_g. PhC cavities were not introduced in this measurement, to examine the optical properties of graphene. A continuous wave (cw) laser diode with a wavelength of 1550 nm and an incident power of 135 μW was used as a light source. Error bars denote one standard deviation from an average of 30 measurements. The absorption of graphene (right *y*-axis, blue circles) was estimated from the measured transmittance. We assumed that the absorption of graphene is 0.023 at V_g = 0.0 V (ref. 3) and its change is the same as the change of transmittance. Also, to estimate the optical loss of graphene, $\kappa_{graphene}$, we compared the relative change of $\kappa_{graphene}$ with the relative change of the absorption of graphene, as we set $\kappa_{graphene}$ = 0.20 THz at V_g = 0.0 V. Then, $\kappa_{graphene}$ = 0.17 and 0.13 THz for V_g = −0.4 and −0.9 V, respectively.

Supplementary Note 1

1. Asymmetric gain model of non-Hermitian coupled cavities

In Hermitian coupled cavities composed of two identical cavities without gain or loss, the eigenfrequencies of supermodes are real and show the broken degeneracy: bonding and anti-bonding modes. However, by introducing gain and loss in the coupled cavities (non-Hermitian), the eigenfrequencies are no longer real. The set of coupled differential equations describing the interplay between the supermodes excited in non-Hermitian coupled cavities is given by

$$i\frac{d}{dt}\begin{bmatrix}\phi_1\\ \phi_2\end{bmatrix} = H\begin{bmatrix}\phi_1\\ \phi_2\end{bmatrix} = 2\pi\begin{bmatrix} f_1 + i(\gamma_1-\kappa_1) & J \\ J & f_2 + i(\gamma_2-\kappa_2)\end{bmatrix}\begin{bmatrix}\phi_1\\ \phi_2\end{bmatrix}, \qquad (1)$$

where H is the effective Hamiltonian of the coupled cavities, J is the coupling constant, and ϕ_1 (ϕ_2), f_1 (f_2), γ_1 (γ_2) and κ_1 (κ_2) are the wave function, eigenfrequency, gain and loss in cavity 1 (cavity 2), respectively[4-6]. In the system of two identical cavities, we set $f_1 = f_2 = f_0$. With the assumption of the supermodes in steady state, the wave functions of supermodes become

$$\begin{bmatrix}\phi_1(t)\\ \phi_2(t)\end{bmatrix} = \begin{bmatrix}\phi_1(0)\\ \phi_2(0)\end{bmatrix}\exp\left[-i2\pi ft\right], \qquad (2)$$

and then, the eigenfrequencies of the supermodes are given by

$$f_\pm = f_0 \pm \sqrt{J^2 - (\Delta\gamma - \Delta\kappa)^2} + i\left(\gamma_{\text{avg}} - \kappa_{\text{avg}}\right), \qquad (3)$$

where $\Delta\gamma = 1/2 \times (\gamma_2 - \gamma_1)$, $\Delta\kappa = 1/2 \times (\kappa_2 - \kappa_1)$, $\gamma_{\text{avg}} = 1/2 \times (\gamma_1 + \gamma_2)$ and $\kappa_{\text{avg}} = 1/2 \times (\kappa_1 + \kappa_2)$ are the gain contrast, loss contrast, average gain and average loss, respectively.

We note that the effective Hamiltonian in Supplementary Eq. 1 is parity–time (PT)-symmetric when the net cavity gain is balanced with the net loss, $(\gamma_1 - \kappa_1) = -(\gamma_2 - \kappa_2)$, or the average cavity gain is balanced with the average loss, $\gamma_{\text{avg}} = \kappa_{\text{avg}}$ (ref. 6). In the PT-symmetric case, the eigenfrequencies are given by

$$f_\pm = f_0 \pm \sqrt{J^2 - (\Delta\gamma - \Delta\kappa)^2}\ . \qquad (4)$$

Supplementary Eq. 4 shows that the exceptional point (EP) occurs at $|\Delta\gamma - \Delta\kappa| = J$. Under balanced gain and loss condition, the unbroken PT-symmetry and the broken PT-symmetry phases occur at $|\Delta\gamma - \Delta\kappa| < J$ and $|\Delta\gamma - \Delta\kappa| > J$, respectively. In the unbroken PT-symmetry phase, the eigenfrequencies are real and the degeneracy is broken with frequency splitting. However, in the broken PT-symmetry phase, the complex eigenfrequencies show frequency

degeneracy in the real part but splitting in the imaginary part. The imaginary part of the eigenfrequencies, Im(f), determines whether supermodes are amplified (Im(f) > 0), sustained (Im(f) = 0), or dissipated (Im(f) < 0)[4-6]. In particular, the supermodes in the broken PT-symmetry phase are either amplified or dissipated, whereas the supermodes in the unbroken PT-symmetry phase exhibit stable oscillations.

More generally, the EP and the phase transition can be observed even under unbalanced gain and loss condition ($\gamma_{avg} - \kappa_{avg} \neq 0$) (see Supplementary Eq. 3). Appropriate asymmetric gain and loss contrasts compared to the coupling constant drive the occurrence of EPs, similarly to the PT-symmetric case, while the average net gain of ($\gamma_{avg} - \kappa_{avg}$) determines the amplification or dissipation of supermodes. In the unbroken PT-symmetry phase ($|\Delta\gamma - \Delta\kappa| < J$), the real part of the eigenfrequencies shows the broken degeneracy, but the imaginary part is coalesced to the average net gain. Conversely, in the broken PT-symmetry phase ($|\Delta\gamma - \Delta\kappa| > J$), the real part of eigenfrequencies is coalesced to f_0, whereas the imaginary part exhibits the broken degeneracy with frequency splitting of $2((\Delta\gamma - \Delta\kappa)^2 - J^2)^{1/2}$ and an offset of the average net gain. These results show that one can circumvent difficulties in satisfying the complicated conditions for the balanced gain and loss in PT-symmetric systems with significant material dispersion[4,7-9].

In the coupled PhC cavities, we assumed that the two identical PhC cavities have the same intrinsic losses, $\kappa_1 = \kappa_2 = \kappa$, including material absorption in the InGaAsP slab and radiation of light from the cavities[9,10]. With the symmetric loss, the eigenfrequencies are given by

$$f_{\pm} = f_0 \pm \sqrt{J^2 - (\Delta\gamma)^2} + i(\gamma_{avg} - \kappa). \qquad (5)$$

Supplementary Eq. 5 explains the gain-dependent features of complex eigenfrequencies in our coupled PhC cavities in Figs. 1b and 1c. J is constant owing to the fixed numbers of PhC air holes between cavities 1 and 2, but $\Delta\gamma$ is varied as the asymmetric gain is applied. The leveraged $\Delta\gamma$ compared to J determines whether the supermodes are in the unbroken PT-symmetry phase ($|\Delta\gamma| < J$) or the broken PT-symmetry phase ($|\Delta\gamma| > J$). The EPs then occur at $|\Delta\gamma| = J$. In Figs. 1b and 1c, by using Supplementary Eq. 5, the calculated complex eigenfrequencies were fitted and the modelling parameters such as f_0, J, and κ were obtained: f_0 = 199.02 THz, J = 0.34 THz, and κ = 0.57 THz.

Lastly, in our coupled cavities with monolayer graphene, we developed the asymmetric gain model similar to Supplementary Eq. 1–5, but the effective gain including the optical loss of

graphene was considered (Figs. 2–5). For example, when the graphene covers only cavity 1, Supplementary Eq. 3 becomes

$$f_{\pm} = f_0 \pm \sqrt{J^2 - \left(\frac{\gamma_2 - \gamma_1}{2} - \frac{\kappa_2 - \kappa_1 - \kappa_{\text{graphene}}}{2}\right)^2} + i\left(\frac{\gamma_1 + \gamma_2}{2} - \frac{\kappa_1 + \kappa_2 + \kappa_{\text{graphene}}}{2}\right), \qquad (6)$$

where κ_{graphene} is the optical loss induced by graphene in cavity 1. Then, we assume the identical intrinsic losses in both cavities, $\kappa_1 = \kappa_2 = \kappa$, and define the effective gain of cavity 1, $\gamma_{1,\text{eff}} = \gamma_1 - \kappa_{\text{graphene}}$. As a result, Supplementary Eq. 6 becomes exactly the same as Supplementary Eq. 5, where $\Delta\gamma = 1/2 \times (\gamma_2 - \gamma_{1,\text{eff}})$ and $\gamma_{\text{avg}} = 1/2 \times (\gamma_{1,\text{eff}} + \gamma_2)$. Therefore, our simple theoretical model using effective gain is useful for identifying the observed lasing modes in non-Hermitian coupled cavities (Fig. 4).

Supplementary References

1. Majumdar, A., Kim, J., Vučković, J. & Wang, F. Electrical control of silicon photonic crystal cavity by graphene. *Nano Lett.* **13**, 515–518 (2013).

2. Thareja, V. *et al.* Electrically tunable coherent optical absorption in graphene with ion gel. *Nano Lett.* **15**, 1570–1576 (2015).

3. Bonaccorso, F., Sun, Z., Hasan, T. & Ferrari, A. C. Graphene photonics and optoelectronics. *Nat. Photonics* **4**, 611–622 (2010).

4. Brandstetter, M. *et al.* Reversing the pump dependence of a laser at an exceptional point. *Nat. Commun.* **5,** 4034 (2014).

5. El-Ganainy, R., Khajavikhan, M. & Ge, L. Exceptional points and lasing self-termination in photonic molecules. *Phys. Rev. A* **90,** 013802 (2014).

6. Ge, L. & El-Ganainy, R. Nonlinear modal interactions in parity-time (PT) symmetric lasers. *Sci. Rep.* **6,** 24889 (2016).

7. Peng, B. *et al.* Loss-induced suppression and revival of lasing. *Science* **346,** 328–332 (2014).

8. Feng, L., Wong, Z. J., Ma, R.-M., Wang, Y. & Zhang, X. Single-mode laser by parity-time symmetry breaking. *Science* **346,** 972–975 (2014).

9. Hodaei, H., Miri, M.-A., Heinrich, M., Christodoulides, D. N. & Khajavikhan, M. Parity-time-symmetric microring lasers. *Science* **346,** 975–978 (2014).

10. Hamel, P. *et al.* Spontaneous mirror-symmetry breaking in coupled photonic-crystal nanolasers. *Nat. Photonics* **9,** 311–315 (2015).
